# Transcriptional signatures of the small intestinal mucosa in response to ethanol in transgenic mice rich in endogenous n3 fatty acids

**DOI:** 10.1038/s41598-020-76959-6

**Published:** 2020-11-16

**Authors:** Josiah E. Hardesty, Jeffrey B. Warner, Ying L. Song, Eric C. Rouchka, Chih-Yu Chen, Jing X. Kang, Craig J. McClain, Dennis R. Warner, Irina A. Kirpich

**Affiliations:** 1 Division of Gastroenterology, Hepatology, and Nutrition, Department of Medicine, University of Louisville, 505 Hancock St., Louisville, KY 40202 USA; 2 Department of Pharmacology and Toxicology, University of Louisville School of Medicine, Louisville, KY USA; 3 Department of Computer Science and Engineering, Speed School of Engineering, University of Louisville, Louisville, KY USA; 4 Laboratory for Lipid Medicine and Technology, Department of Medicine, Massachusetts General Hospital and Harvard Medical School, Boston, MA USA; 5 University of Louisville Alcohol Center, University of Louisville School of Medicine, Louisville, KY USA; 6 University of Louisville Hepatobiology and Toxicology Center, University of Louisville School of Medicine, Louisville, KY USA; 7 Robley Rex Veterans Medical Center, Louisville, KY USA

**Keywords:** Alcoholic liver disease, Nutrition, Fatty acids

## Abstract

The intestine interacts with many factors, including dietary components and ethanol (EtOH), which can impact intestinal health. Previous studies showed that different types of dietary fats can modulate EtOH-induced changes in the intestine; however, mechanisms underlying these effects are not completely understood. Here, we examined intestinal transcriptional responses to EtOH in WT and transgenic *fat-1* mice (which endogenously convert n6 to n3 polyunsaturated fatty acids [PUFAs]) to identify novel genes and pathways involved in EtOH-associated gut pathology and discern the impact of n3 PUFA enrichment. WT and *fat-1* mice were chronically fed EtOH, and ileum RNA-seq and bioinformatic analyses were performed. EtOH consumption led to a marked down-regulation of genes encoding digestive and xenobiotic-metabolizing enzymes, and transcription factors involved in developmental processes and tissue regeneration. Compared to WT, *fat-1* mice exhibited a markedly plastic transcriptome response to EtOH. Cell death, inflammation, and tuft cell markers were downregulated in *fat-1* mice in response to EtOH, while defense responses and PPAR signaling were upregulated. This transcriptional reprogramming may contribute to the beneficial effects of n3 PUFAs on EtOH-induced intestinal pathology. In summary, our study provides a reference dataset of the intestinal mucosa transcriptional responses to chronic EtOH exposure for future hypothesis-driven mechanistic studies.

## Introduction

The gastrointestinal (GI) tract interacts dynamically with a variety of environmental factors, including different dietary components and ethanol (EtOH). Dietary fats are among the most important factors contributing to intestinal homeostasis and basic functions, and are tightly regulated in order to maintain intestinal health and overall well-being^1,2^. Dietary nutrients can also shape the GI microbiota and intestinal immunity^3^. Therefore, a well-balanced diet is crucial for good health. However, in the Western diet, n6 polyunsaturated fatty acid (PUFA) consumption has become progressively higher than n3 PUFA consumption, and this unbalanced diet may have a negative impact on health and multiple pathological conditions. An increase in the n6/n3 PUFA ratio has been shown to be detrimental in multiple chronic diseases^4,5^. It is well recognized that dietary PUFAs play an important role in intestinal health. Reduced intestinal inflammation by n3 PUFA supplementation has been shown both in patients and in animal models, suggesting anti-inflammatory and pro-resolving properties of n3 PUFAs and their derivatives in the gut (reviewed in^6^). EtOH consumption, in general, negatively affects the GI tract causing damaging effects on intestinal health in experimental animals and in humans. These effects include pathological changes in numerous metabolic pathways and functions such as loss of gut barrier integrity, increased inflammation, and microbial dysbiosis, among others^7–9^. Notably, composition of dietary fats can modulate (either exacerbate or attenuate) EtOH-induced changes in the intestine. It has been demonstrated that EtOH and a diet enriched in corn oil (containing a high amount of the n6 PUFA linoleic acid), but not a diet enriched in medium chain fatty acids (MCFAs), results in increased gut permeability, alterations in the intestinal mucus layer and antimicrobial defense, and an increased intestinal pro-inflammatory response^8,10^. Supplementation with saturated long-chain fatty acids (LCFAs) positively affected EtOH-mediated alterations in the intestinal barrier and gut microbiota dysbiosis^11^. It has been shown that tributyrin, a triglyceride found in butter and margarine, prevents alcohol-induced tight-junction (TJ) disruption, which in turn protects against intestinal hyperpermeability^12^. Various dietary fatty acids also differentially modulate EtOH-induced changes in the gut microbiome. For example, EtOH in combination with n6 PUFAs, but not MCFAs, causes an increase in *Proteobacteria* and *Actinobacteria*^13^.

A recent study from our group demonstrated that the increase in endogenous n3 PUFAs and subsequent decrease in the n6/n3 PUFA ratio in transgenic *fat-1* mice (which endogenously convert n6 to n3 PUFAs) attenuated EtOH-mediated alterations in the gut-liver axis^14^. Specifically, we found protection against EtOH-mediated downregulation of intestinal TJ proteins ex vivo in organoid cultures and in vivo in *fat-1* mice. Further, although EtOH feeding did not cause marked structural damage to the ileum in either WT or *fat-1* mice, expression of markers of intestinal stem cells growth and proliferation were higher in ileal organoids obtained from *fat-1 *vs WT animals. In addition, compared to WT, *fat-1* mice developed less intestinal inflammation in response to EtOH and lipopolysaccharide (LPS) challenge. Thus, the types of dietary fat that reach the intestine and the endogenous tissue levels of n3 and n6 PUFAs are critical modulators of EtOH-associated gut abnormalities. Although recent evidence supports the beneficial effects of n3 PUFAs on EtOH-mediated alterations in intestinal homeostasis^14^, the molecular mechanisms underlying these effects are not completely understood. The current study is an extension of our previous work analyzing the intestinal transcriptional responses to chronic EtOH administration that addresses the following goals: (1) to identify novel genes and pathways of intestinal dysregulation that contribute to alcohol-associated gut pathology; and (2) to examine gene expression signatures underlying the beneficial effects of n3 PUFA-mediated intestinal protection against EtOH-induced damage. Understanding changes in the intestinal transcriptome and how these changes influence host metabolism/health in vivo may identify novel therapeutic targets for the treatment of alcohol-induced intestinal pathology.

## Results

### Characteristics of the animal model and overview of intestinal RNAseq analysis

To examine the impact of chronic EtOH administration and elevated levels of endogenous n3 PUFAs on the intestinal transcriptome, we performed RNAseq analysis and compared the transcriptomes of intestinal epithelial tissue obtained from WT and *fat-1* mice subjected to control (pair-fed mice, PF) or EtOH-containing diets (EtOH-fed mice, see Supplemental Fig. S1a for study design). Animal daily food consumption and body weights can be found in Supplemental Fig. S1b,c, respectively. While there were no differences in food consumption between genotypes, as well as between PF and EtOH-fed animals, WT EtOH, WT PF, and *fat-1* PF mice gained body weight by the end of the experiment, whereas *fat-1* EtOH mice did not. Compared to WT*, fat-1* animals exhibited elevated intestinal tissue n3 PUFA levels in both PF and EtOH-fed mice (see ref.^14^ and Supplemental Fig. S1d). There were no differences in n6 PUFAs between WT and *fat-1* control PF mice; however, elevated intestinal n6 PUFA levels were observed in response to EtOH in WT but not in *fat-*1 littermates. In addition, the ratio of n6/n3 PUFAs was significantly lower in *fat-1* compared to WT mice in both PF and EtOH-fed groups.

Intestinal RNAseq analysis performed on these mice revealed that both the genotype and EtOH administration caused alterations in the intestinal transcriptome; the full list of significant differentially expressed genes (DEGs) can be found in Supplemental Materials Excel File S1. We next applied a cutoff of ≥ two-fold change for the DEGs used in Volcano Plots, GO Processes, and STRING Cluster Analyses. This threshold was used to identify robust gene expression changes and the accompanying biological processes. There were 630 DEGs between *fat-1* PF and WT PF animals (420 up and 210 down). Of those genes, 276 DEGs between *fat-1* PF and WT PF animals met the two-fold change threshold for further analysis (193 up and 83 down). EtOH administration resulted in 1144 DEGs (481 up and 663 down) in the WT EtOH vs WT PF group, and of these genes, 439 met the two-fold change threshold (163 up and 276 down). While 2107 genes were differentially expressed in *fat-1* EtOH vs* fat-1* PF mice (1045 up and 1062 down), 1501 met the two-fold change threshold for further analyses (812 up and 689 down). 94 genes were significantly different (80 up and 14 down) in the WT EtOH group when compared to *fat-1* EtOH mice (all of these DEGs met the two-fold change threshold).

### Differentially expressed genes between *fat-1* and WT control pair-fed animals

**Figure 1:**
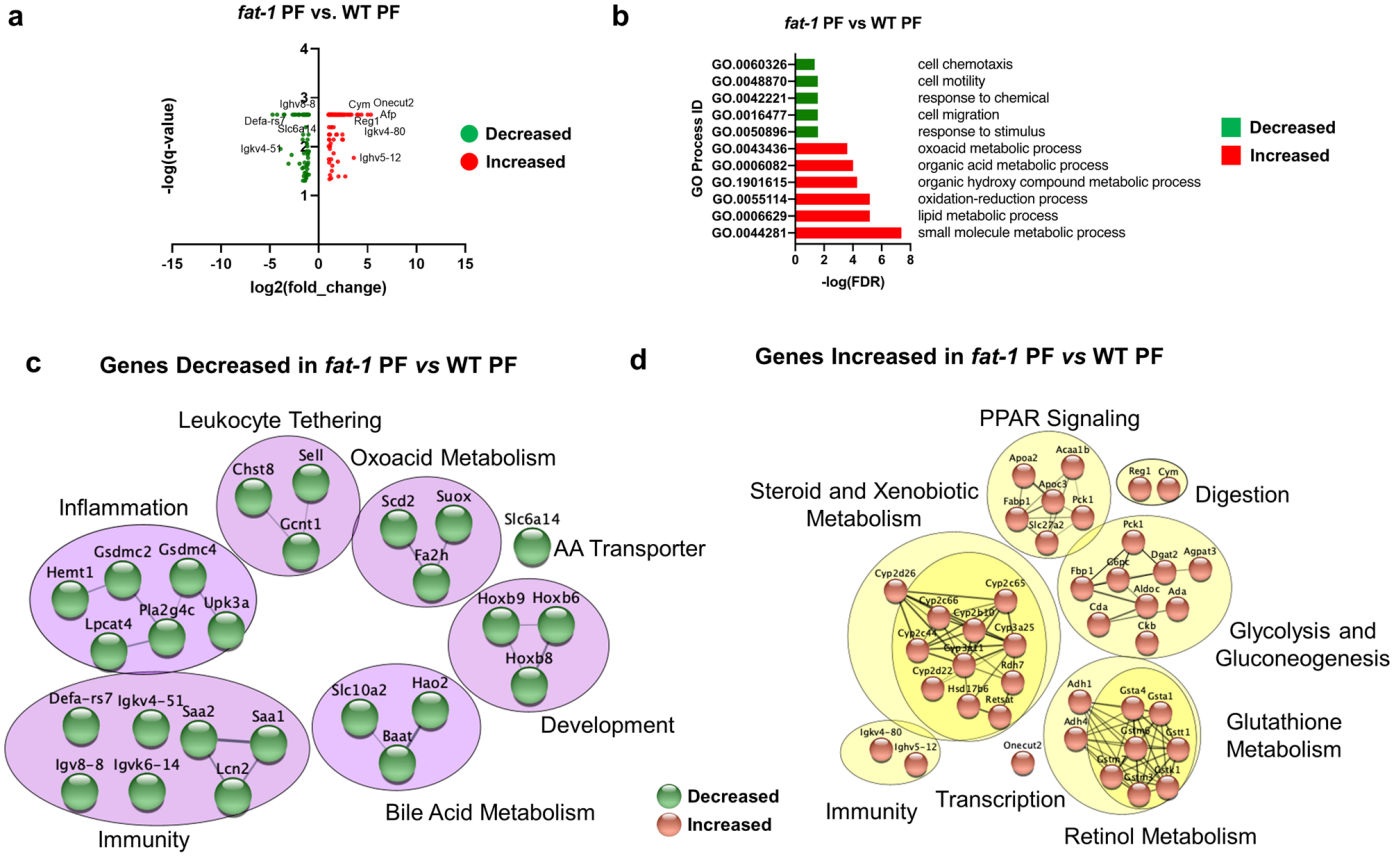
Differentially expressed genes between *fat-1* and WT control pair-fed animals. (**a**) Volcano plot analysis of significant genes from the *fat-1* PF vs WT PF comparison. Genes are plotted as log_2_(fold change) vs antilog(p-value). (**b**) GO Processes representative of decreased gene expression (green) and increased gene expression (red) for the *fat-1* PF vs WT PF comparison. (**c**) Gene cluster analysis of genes decreased in *fat-1* PF vs WT PF comparison (purple circles, green nodes). (**d**) Gene cluster analysis of genes increased in *fat-1* PF vs WT PF comparison (yellow circles, copper nodes). Significant DEGs were selected based on a q-value < 0.05. Significant DEGs with fold-change greater than or equal to two-fold were used for analyses in (**a**–**d**). n = 3–5 mice per group.

The most prominent changes among genes differentially expressed in *fat-1* PF compared to WT PF mice (≥ two-fold change, Fig. 1a and Supplementary Table S1) were observed for *Onecut2, Reg1, Igkv4-80, Cym, Ighv5-12, and Afp* (increased expression), and *Defa-rs7, Igk4-51, Slc6a14*, and *Ighv8-8,* (decreased expression). In order to best classify *fat-1* PF vs WT PF mice with respect to all DEGs that met the two-fold threshold, we performed Gene Ontology (GO) pathway analysis in CytoScape. The top enriched and diminished pathways are shown in Fig. 1b. Next, gene clusters identified by STRING were used to visualize gene interactions and identify processes affected by the endogenous increase in n3 PUFAs in *fat-1* compared to WT mice. The gene and protein interactions inferred from differentially expressed transcripts between *fat-1* PF and WT PF included down-regulated gene networks (Fig. 1c) for leukocyte tethering (*Chst8, Gcnt1, Sell*), inflammation (*Gsdmc2, Gsdmc4, Pla2g4c*), immunity (*Saa2, Saa1, Lcn2*), bile acid metabolism (*Slc10a2, Hao2, Baat*), development (*Hoxb9, Hoxb6, Hoxb8*), oxoacid metabolism (*Scd2, Suox2, Fa2h*), and amino acid transport (*Slc6a14*). Up-regulated gene networks (Fig. 1d) included metabolism of steroids/xenobiotics (*Cyp2c65, Cyp2b10, Cyp2b66*), glycolysis and gluconeogenesis (*Fbp1, G6pc, Pck1*), glutathione metabolism (*Gstt1, Gstm3, Gsta1*), peroxisome proliferator-activated receptor (PPAR) signaling (*Apoa2, Fabp1, Acaa1b*), immunity (*Igkv4-80*, *Ighv5-12*), digestion (*Reg1, Cym*), and transcription (*Onecut2*).

### EtOH-mediated alterations in the intestinal transcriptome: similarity in transcriptional responses between WT and *fat-1* mice

**Figure 2:**
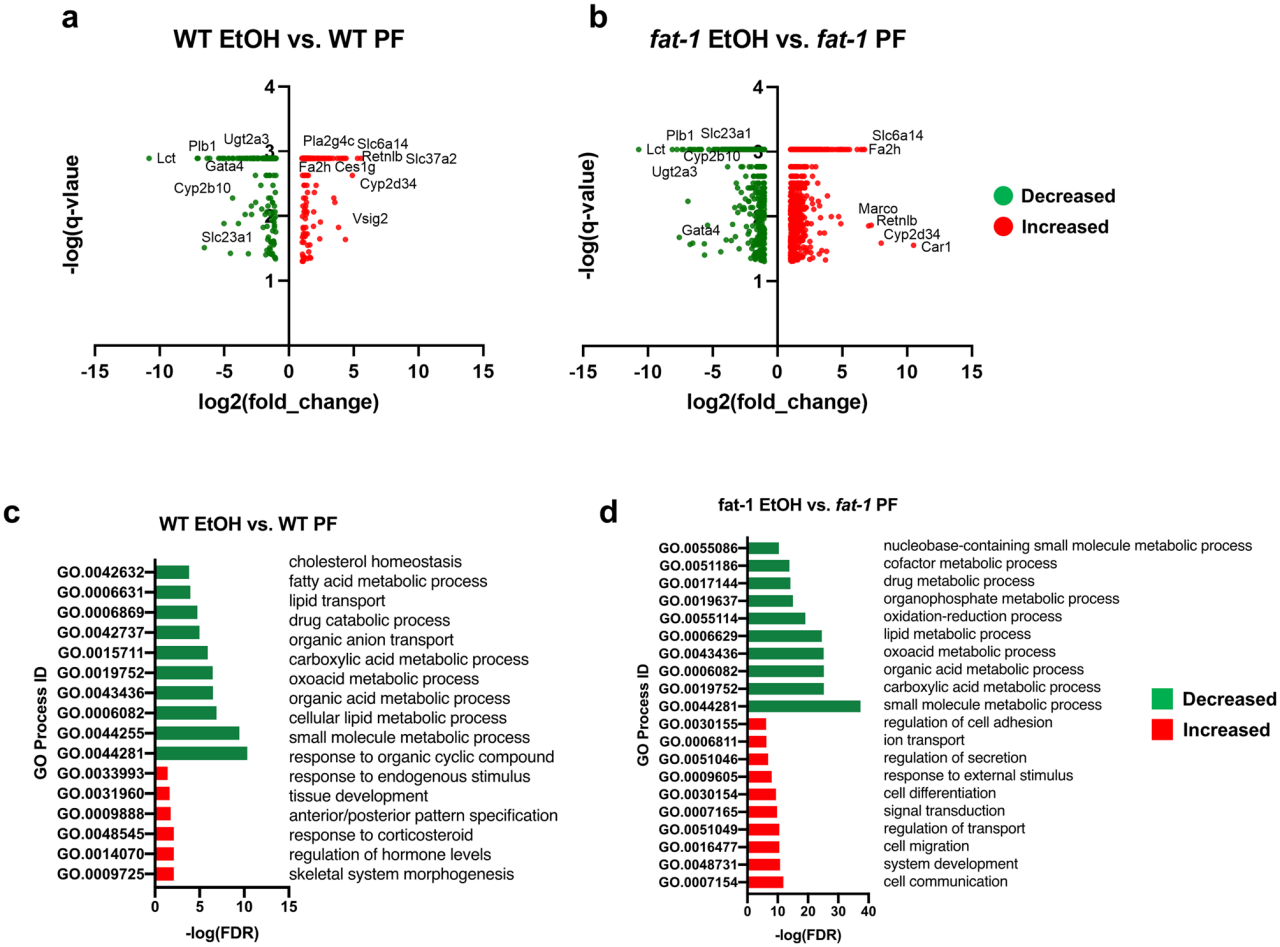
Changes in intestinal transcriptional responses to EtOH in WT and *fat-1* mice. (**a**) Volcano plot analysis of significant genes from the WT EtOH vs WT PF comparison plotted as log_2_(fold change) vs antilog(p-value) for individual genes. (**b**) Volcano plot analysis for the *fat-1* EtOH vs* fat-1* PF comparison plotted as log_2_(fold change) vs antilog(p-value) for individual genes. (**c**) GO Process analysis representative of genes decreased in expression (green) and genes increased in expression (red) for the WT EtOH vs WT PF comparison. (**d**) GO Process analysis representative of genes decreased in expression (green) and genes increased in expression (red) for the *fat-1* EtOH vs* fat-1* PF comparison. Significant DEGs that met a two-fold threshold were used for the analyses in (**a**–**d**). n = 3–5 mice per group.

EtOH administration resulted in substantial alterations in intestinal gene expression. Notably, there were almost 2 times more genes differentially expressed in *fat-1* mice compared to WT littermates in response to EtOH (2107 DEGs vs 1144 DEGs, respectively), suggesting that the alterations in the associated lipid profile and, possibly, lipid homeostasis in *fat-1* mice resulted in elevated gene transcription changes in response to EtOH challenge. Further analysis revealed a set of 835 genes in WT and *fat-1* mice with similarly changed expression in response to EtOH (321 ≥ two-fold change), while 1272 and 309 genes were exclusively changed by EtOH in *fat-1* and WT mice, respectively (180 and 118 ≥ two-fold change). Volcano plot analysis of DEGs that met the two-fold threshold in WT-EtOH relative to WT-PF (Fig. 2a) and in *fat-1* EtOH relative to *fat-1* PF (Fig. 2b) demonstrated that the top down-regulated genes shared by both genotypes included *Lct, Cyp2b10, Ugt2a3, Gata4,* and *Plb1*. Among the genes up-regulated by EtOH in both WT and *fat-1* mice were *Cyp2d34, Retnlb, Slc6a14,* and *Fa2h.* The top DEGs that changed their expression in response to EtOH in WT and *fat-1* mice, as well as shared genes between WT and *fat-*1 mice in response to EtOH are listed in Supplementary Tables S2, S3, and S4, respectively. Cytoscape GO analysis identified downregulated processes in WT-EtOH vs WT-PF mice including lipid metabolism, oxoacid metabolism, anion transport, fatty acid metabolism, and cholesterol homeostasis; while upregulated processes included development and response to hormones (Fig. 2c). GO processes diminished in *fat-1* EtOH vs* fat-1* PF mice included oxoacid metabolism, lipid metabolism, drug metabolism, and nucleobase metabolism, while cell communication, cell migration, signal transduction, transport, cell adhesion, and cell differentiation were enriched (Fig. 2d).

**Figure 3:**
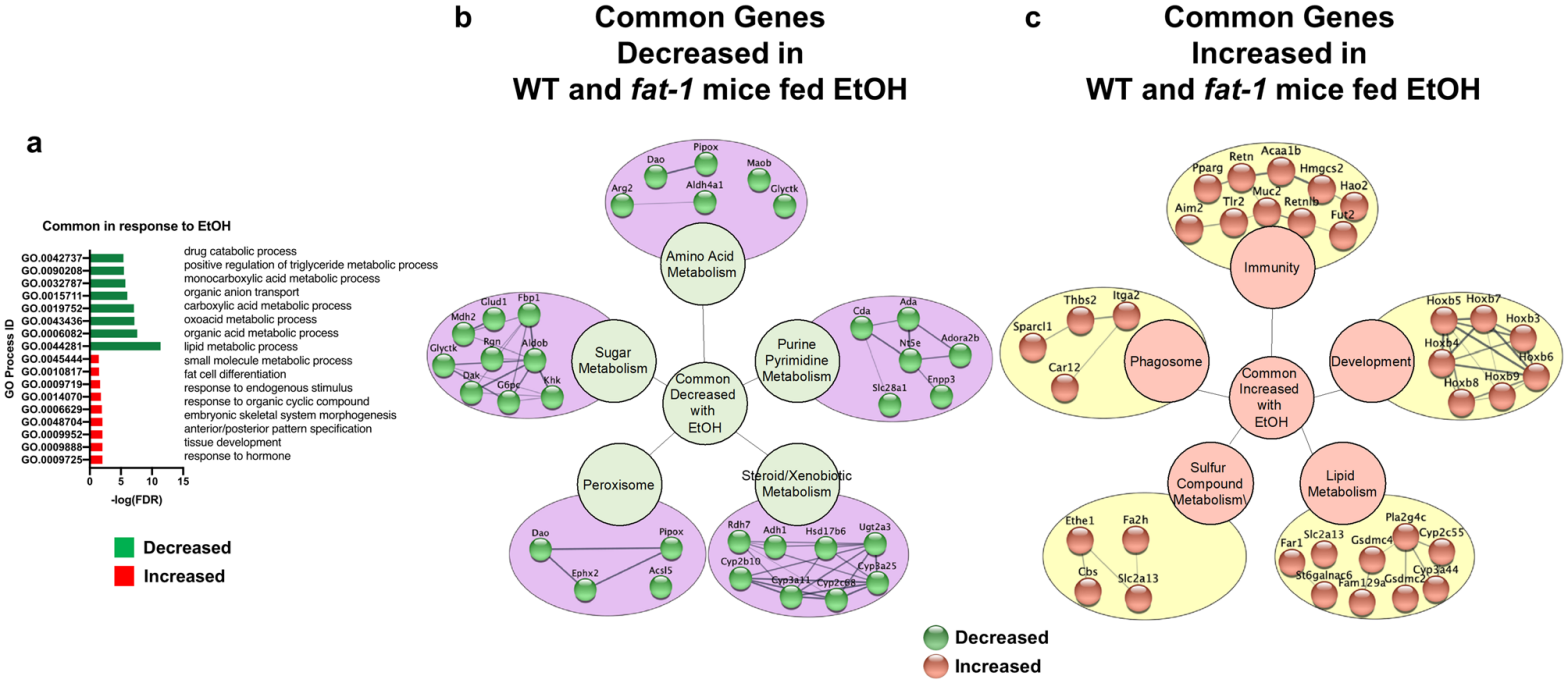
Similarity in transcriptional responses of ileum tissue to EtOH in WT and *fat-1* mice. (**a**) GO Process analysis representative of genes decreased in expression (green) and genes increased in expression (red) that were shared between genotypes in response to EtOH. (**b**) Gene cluster analysis of genes shared between genotypes that were decreased in response to EtOH (purple circles, green nodes). (**c**) Gene cluster analysis of genes shared between genotypes that were increased in response to EtOH (yellow circles, copper nodes). Significant DEGs that met a two-fold change threshold were used for the analyses in (**a**–**c**). n = 3–5 mice per group.

GO processes representative of the decreased genes in response to EtOH shared between genotypes included lipid metabolism, carboxylic acid metabolism, drug metabolism, and anion transport. The elevated shared genes due to EtOH included hormone and developmental processes (Fig. 3a). Gene clusters identified by STRING were used to visualize gene interactions and pathways among DEGs commonly affected by EtOH in both genotypes. Common pathway clusters decreased by EtOH in WT and *fat-*1 mice included amino acid metabolism (*Arg2*, *Dao*, *Pipox*), sugar metabolism (*Aldob*, *G6pc*, *Fbp1*), purine and pyrimidine metabolism (*Ada*, *Cda, Nt5e*), steroid and xenobiotic metabolism (*Rdh7*, *Adh1*, *Cyp2b10*), and the peroxisome (*Ephx2*, *Dao, Acsl5*) (Fig. 3b). Pathway clusters representative of common genes elevated by EtOH in WT and *fat-1* mice included immunity (*Pparγ*, *Muc2*, *Tlr2*), development (*Hoxb4*, *Hoxb8*, *Hoxb5*), lipid metabolism (*Cyp2c55*, *Cyp3a44*, *Far1*), sulfur compound metabolism (*Cbs*, *Ethe1*, *Fa2h*), and the phagosome (*Itga2*, *Thbs2*, *Sparcl1*) (Fig. 3c).

### EtOH-mediated alterations in the intestinal transcriptome: unique transcriptional responses to EtOH in WT or *fat-1* mice

Next, we identified the specific transcriptional responses to EtOH in the intestinal mucosa of WT and *fat-1* mice. There were a set of genes most prominently up and down-regulated in response to EtOH treatment exclusively in WT but not in *fat-1* mice including *Slc37a2, Ighv14-4, Ighv1-63, Abca12,* and *Rn7sk* (~ 47-, 15-, 14-, 13-, and 11- fold increases, respectively), and *Fabp1, Ighv2-3, Igkv4-51, Gip,* and *Ighv8-8* (~ 70-, 16-, 15-, 14-, and 8- fold decreases, respectively, Supplementary Table S5). The genes most highly changed in response to EtOH treatment exclusively in *fat-1* but not in WT mice included*: Car1, Marco, Nov, Plet1,* and *Cdhr1* (~ 1454-, 151-, 97-, 31-, and 30-fold increases, respectively), and *Bbox1, Slc4a5, Dnah2, Rec8,* and *Afp* (~ 59-, 25-, 20-, 19-, and 16-fold decreases, respectively, Supplementary Table S6).

**Figure 4:**
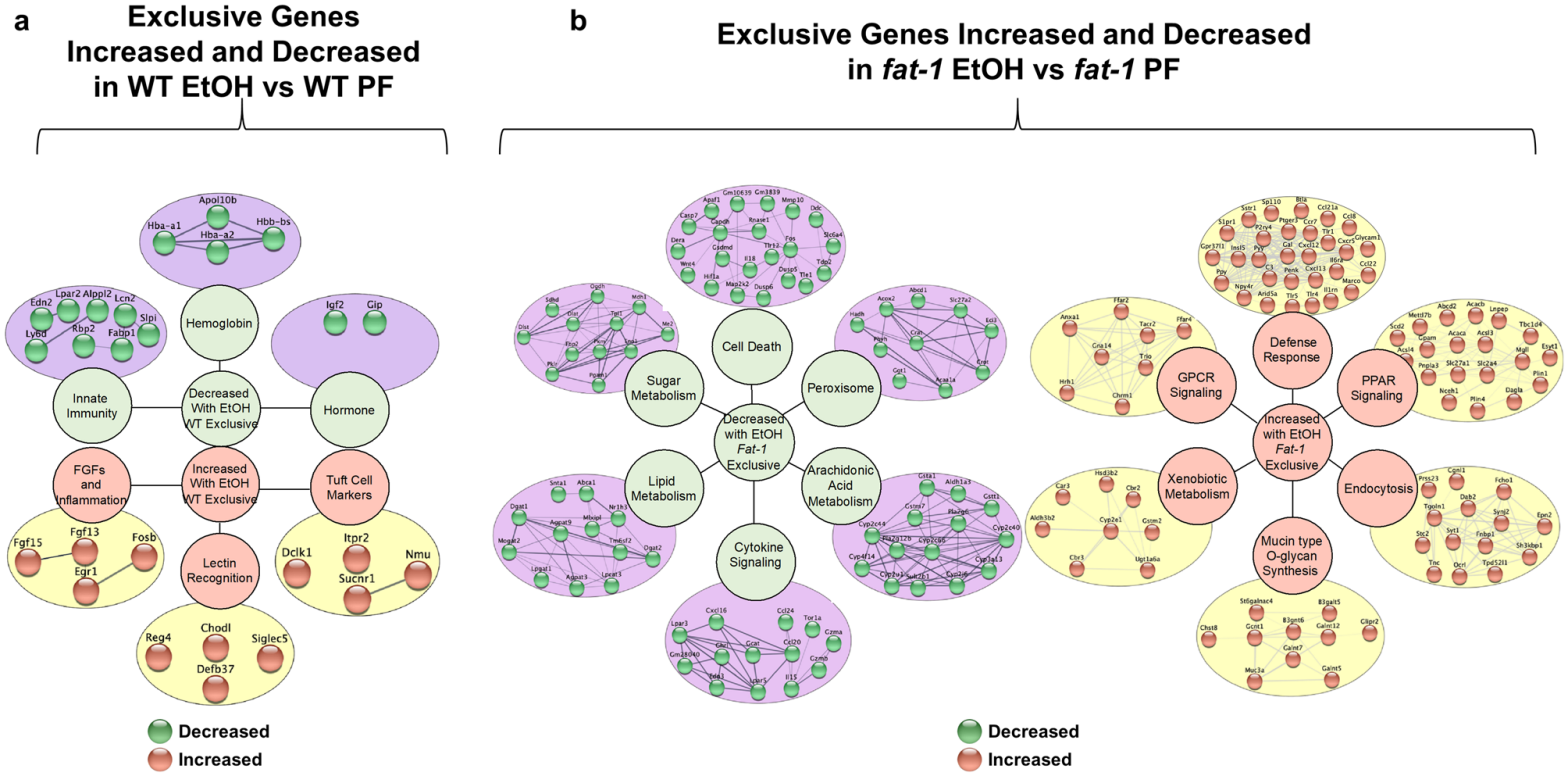
Genotype exclusive transcriptional responses to EtOH in WT and *fat-1* mice. (a) Cluster analysis of genes exclusively decreased (purple circles, green nodes) or increased in response to EtOH in WT mice (yellow circles, copper nodes). (**b**) Cluster analysis of genes exclusively decreased (purple circles, green nodes) or increased in response to EtOH in *fat-1* mice (yellow circle, copper nodes). Significant DEGs that met a two-fold change threshold were used for the analyses in (**a**,**b**). n = 3–5 mice per group.

Cytoscape cluster analysis identified genotype-specific pathway alterations in response to EtOH. Pathways that were downregulated in response to EtOH in WT mice exclusively included hormones (*Igf2*, *Gip*), hemoglobin (*Hba-a1*, *Hba-a2*, *Hbb-bs*), and innate immunity (*Lcn2*, *Slpi*, *Ly6d*). Pathway clusters upregulated in response to EtOH in WT mice only included tuft cell markers (*Itpr2*, *Dclk1*, *Sucnr1*), lectin recognition (*Chodl*, *Siglec5*, *Reg4*), and fibroblast growth factors (FGFs) (*Fgf13*, *Fgf15*) and inflammation (*Fosb*, *Egr1*) (Fig. 4a). Clustered pathways decreased in response to EtOH in *fat-1* mice exclusively include cell death (*Gsdmd*, *Il18*, *Casp7*), the peroxisome (*Hadh*, *Crat*, *Acaa1a*), arachidonic acid metabolism (*Cyp2c66*, *Cyp2j6*, *Cyp2u1*), cytokine signaling (*Il15*, *Cxcl16*, *Gzma*), lipid metabolism (*Nr1h3*, *Dgat1*, *Dgat2*), and sugar metabolism (*Pklr*, *Eno1*, *Mdh1*) (Fig. 4b). Pathway clusters increased in response to EtOH in *fat-1* mice only included defense response (*Marco*, *Il1rn*, *Tlr4*), PPAR signaling (*Pnpla3*, *Plin4*, *Plin1*), endocytosis (*Syt1*, *Dab2*, *Epn2*), mucin O-glycan synthesis (*B3gnt6*, *Gcnt1*, *Gaint12*), xenobiotic metabolism (*Gstm2*, *Cbr2*, *Cbr3*), and GPCR signaling (*Ffar2*, *Ffar4*, *Tacr2*) (Fig. 4b).

### Differential transcriptional responses to EtOH between *fat-1* and WT mice

**Figure 5:**
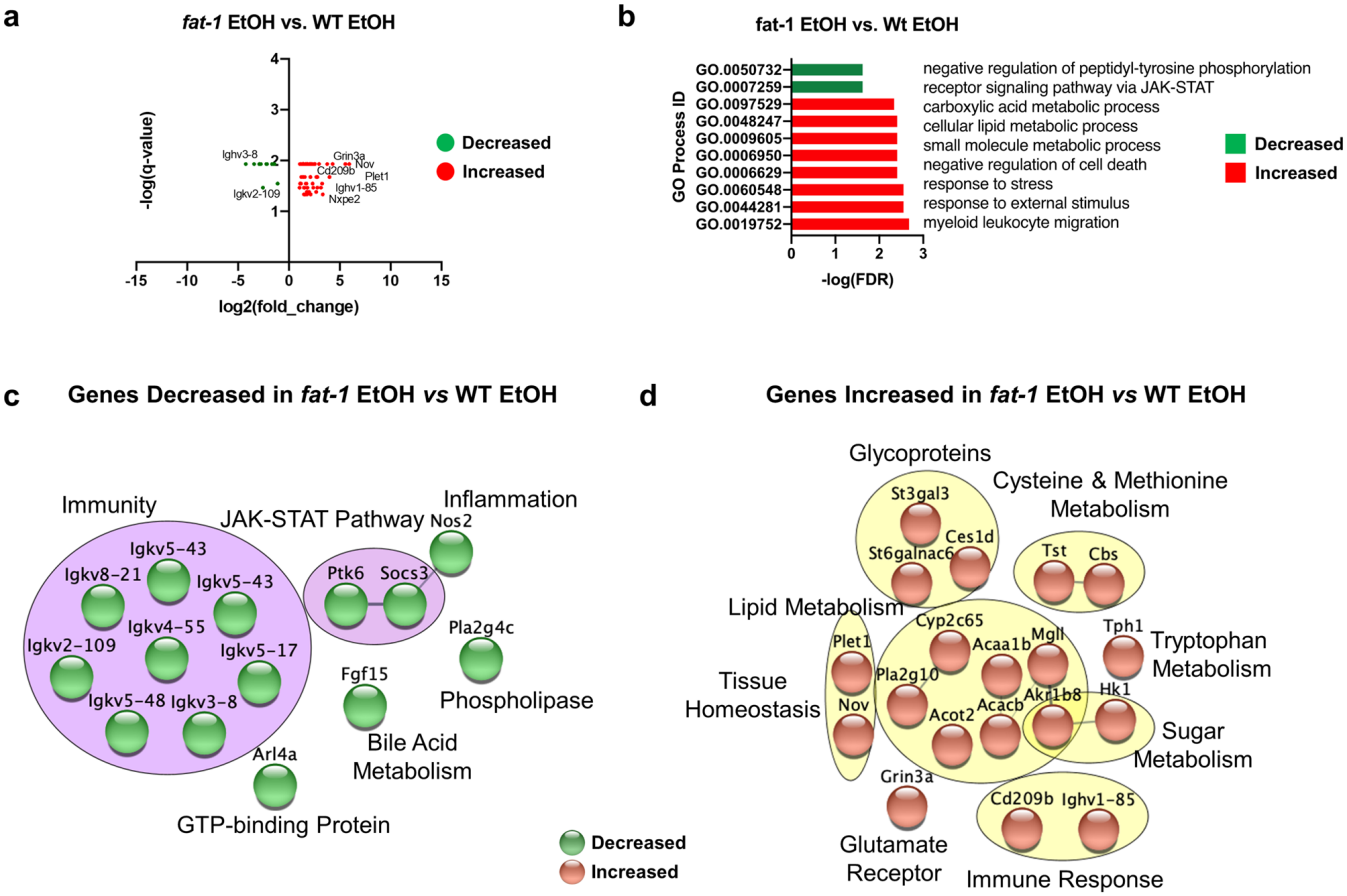
Differential transcriptional responses in *fat-1* EtOH vs WT EtOH mice. (**a**) Volcano plot analysis of significant genes from the *fat-1* EtOH vs WT EtOH comparison plotted as log_2_(fold change) vs. log(p-value) for individual genes. (**b**) GO Process analysis representative of genes decreased in expression (green) and genes increased in expression (red) from the *fat-1* EtOH vs WT EtOH comparison. (**c**) Gene cluster analysis of genes decreased in the *fat-1* EtOH vs WT EtOH comparison (purple circles, green nodes). (**d**) Gene cluster analysis of genes increased in the *fat-1* EtOH vs WT EtOH comparison (yellow circles, copper nodes). Significant DEGs that met a two-fold change threshold were used for the analyses in (**a**–**d**). n = 3–5 mice per group.

The differences described above in transcriptional responses to EtOH in *fat-1* and WT mice resulted in a total of 94 genes that were differentially regulated between *fat-1* EtOH and WT EtOH animals. Among 80 up-regulated DEGs in *fat-1* EtOH vs WT EtOH, the greatest increase was observed for *Nov*, *Plet1, Cd209b, Ighv1-85, Grin3a, Nxpe2* (~ 61-, 46-, 20-, 16-, 14-, and tenfold increases, respectively, Fig. 5a, Supplementary Table S7). Immunoglobulins, including *Ighv3-8 and Igkv2-109* were among the 14 most decreased DEGs in *fat1-1* EtOH vs WT EtOH mice. Processes representative of genes decreased in *fat-1 *EtOH mice included negative regulation of tyrosine phosphorylation and JAK-STAT signaling, while leukocyte migration, stress response, negative regulation of cell death, and lipid metabolism were increased (Fig. 5b). Gene clusters identified by STRING analysis and the pathways they represent were used to visualize ileum DEGs between *fat-*1 EtOH and WT EtOH mice. Clustered pathways downregulated in *fat-1* EtOH vs WT EtOH mice included immunity (*Igkv8-21, Igkv2-109, Igkv5-17*), the JAK-STAT pathway (*Ptk6, Socs3*), inflammation (*Nos2*), GTP-binding protein (*Arl4a*), bile acid metabolism (*Fgf15*), and phospholipase (*Pla2g4c*) (Fig. 5c). Pathway clusters upregulated in *fat-1* EtOH vs WT EtOH mice included glycoproteins (*St3gal3, St6galnac6, Ces1d*), lipid metabolism (*Acaa1b, Cyp2c65, Acacb*), cysteine-methionine metabolism (*Tst, Cbs*), tryptophan metabolism (*Tph1*), sugar metabolism (*Hk1, Akr1b8*), immune response (*Cd209b, Ighv1-85),* glutamate receptor (*Grin3a*), and tissue homeostasis (*Plet1, Nov*) (Fig. 5d).

### Targeted analysis of selected intestinal metabolic pathways relevant to diet and EtOH-mediated alterations of gut homeostasis and overall well-being

#### Effects of EtOH on the expression of free fatty acid receptors in WT and *fat-1* mice

**Figure 6:**
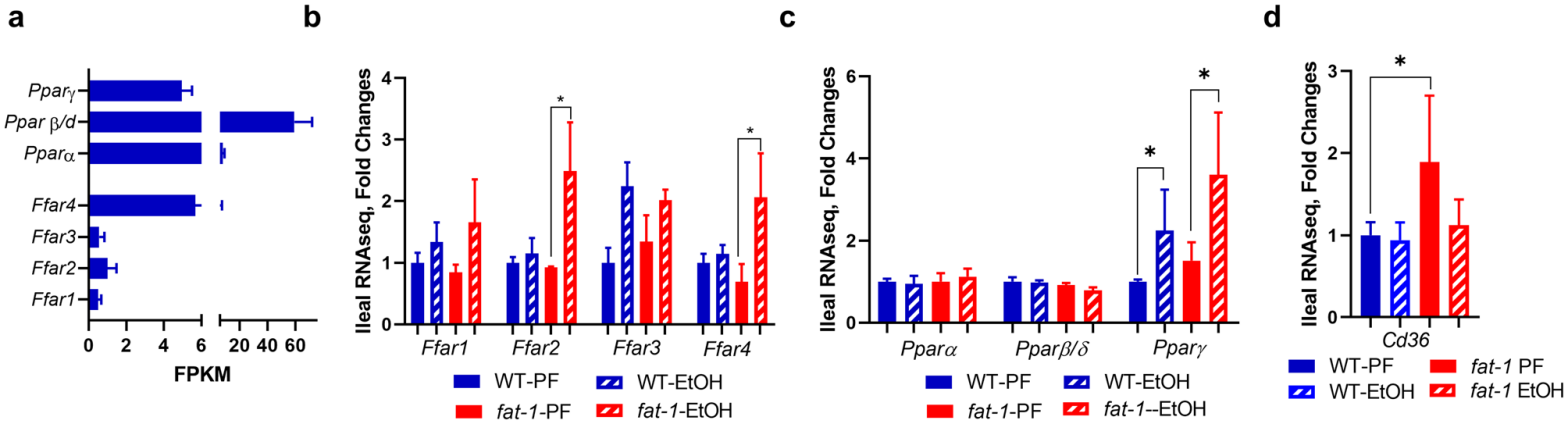
Expression of free fatty acid receptors in *fat-1 *vs WT mice. (**a**) Relative gene expression of FFA receptors (*Ppars* and *Ffars*) in ileal mucosa from WT PF mice. (**b**) Gene expression of *Ffars* in ileal mucosa among all treatment groups. (**c**) Gene expression of *Ppars* in ileal mucosa among all treatment groups. (**d**) Gene expression of *Cd36* in ileal mucosa among all treatment groups. Data are presented as mean ± SEM for FPKM (**a**) and fold changes (**b**–**d**). *p < 0.05. n = 3–5 mice per group.

Nutrient sensing in the gut epithelium is fundamental for intestinal health. Since dietary fatty acids (FAs) can impact intestinal homeostasis, and n3 and n6 PUFAs act as signaling molecules, we were interested in evaluating the expression of receptors recognizing these molecules, including the FFAR and PPAR families. We observed that *Pparβ/δ* was the most abundantly expressed among all FA receptors (Fig. 6a). *Ffar4*, which recognizes MCFAs and LCFAs^15,16^, had the highest expression among the FFARs in the intestinal mucosa (Fig. 6a). There were no evident differences in FA receptors between WT PF and *fat-1* PF mice. EtOH administration resulted in elevated levels of *Ffar2* and *Ffar4* in *fat-1* but not WT mice, and an increase in *Pparγ*, which recognizes LCFAs and LCFA metabolites, in both WT and *fat-1* EtOH-fed animals (Fig. 6b,c). The expression of *Cd36*, which mainly recognizes LCFAs was significantly elevated in *fat-1 *vs WT PF but not EtOH-fed animals (Fig. 6d).

#### Changes in the expression of microbial sensing genes in WT and *fat-1* mice exposed to EtOH

**Figure 7:**
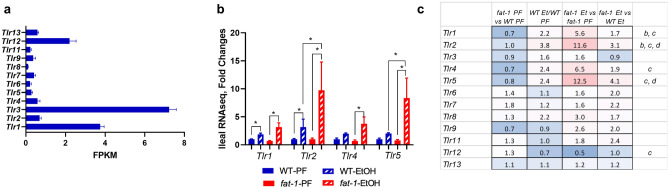
Changes in the expression of microbial sensing genes in WT and *fat-1* mice exposed to EtOH. (**a**) Relative gene expression of *Tlr* genes in ileal mucosa from WT PF mice. Data are presented as mean ± SEM for FPKM. (**b**) Gene expression of *Tlr* genes in ileal mucosa from all treatment groups. Data are expressed as fold changes vs WT PF set as 1, * p < 0.05. (**c**) Heatmap analysis of ileal mucosa *Tlr* genes across multiple comparisons. Data are expressed as fold changes for indicated groups. Significance for the *fat-1* PF vs WT PF comparison is denoted by (**a**), for the WT EtOH vs WT PF (**b**), for *fat-1* EtOH vs* fat-1* PF (**c**), and for *fat-1* EtOH vs WT EtOH (**d**). n = 3–5 mice per group.

The intestinal epithelium expresses a range of pattern recognition receptors (PRRs) that sense and respond to a variety of microbial signals to maintain mucosal homeostasis^17^. Among the PRRs, toll-like receptors (TLRs) are key players in microbe and microbial-product recognition. TLRs are also significantly involved in host defense and tissue repair responses^18^, and play a critical role in EtOH-mediated immune response^19^. In addition, saturated and unsaturated FAs may exert their effects via activation of TLRs like TLR2 and TLR4^20^. In our study, we observed that *Tlr3* was the highest expressed TLR in the intestinal mucosa, followed by *Tlr1* and *Tlr12* (Fig. 7a). Further, there was a significant increase in *Tlr1* and *Tlr2* expression in both *fat-1* and WT EtOH-fed mice compared to control PF animals, while *Tlr4* and *Tlr5* were significantly up-regulated by EtOH only in *fat-1* mice. Interestingly, the expression of *Tlr2* and *Tlr5* were significantly higher in *fat-1* EtOH compared to WT EtOH (Fig. 7b). *Tlr12* was the only family member decreased by EtOH in *fat-1* mice (Fig. 7c). There were no significant differences between any experimental groups in the expression of other *Tlrs* or other PRR families, *e.g.,* nucleotide-binding oligomerization domain-like receptors (*Nlrs*, data not shown).

#### Impact of EtOH on the intestinal genes involved in the adenosine signaling pathway in WT and *fat-1* mice

**Figure 8:**
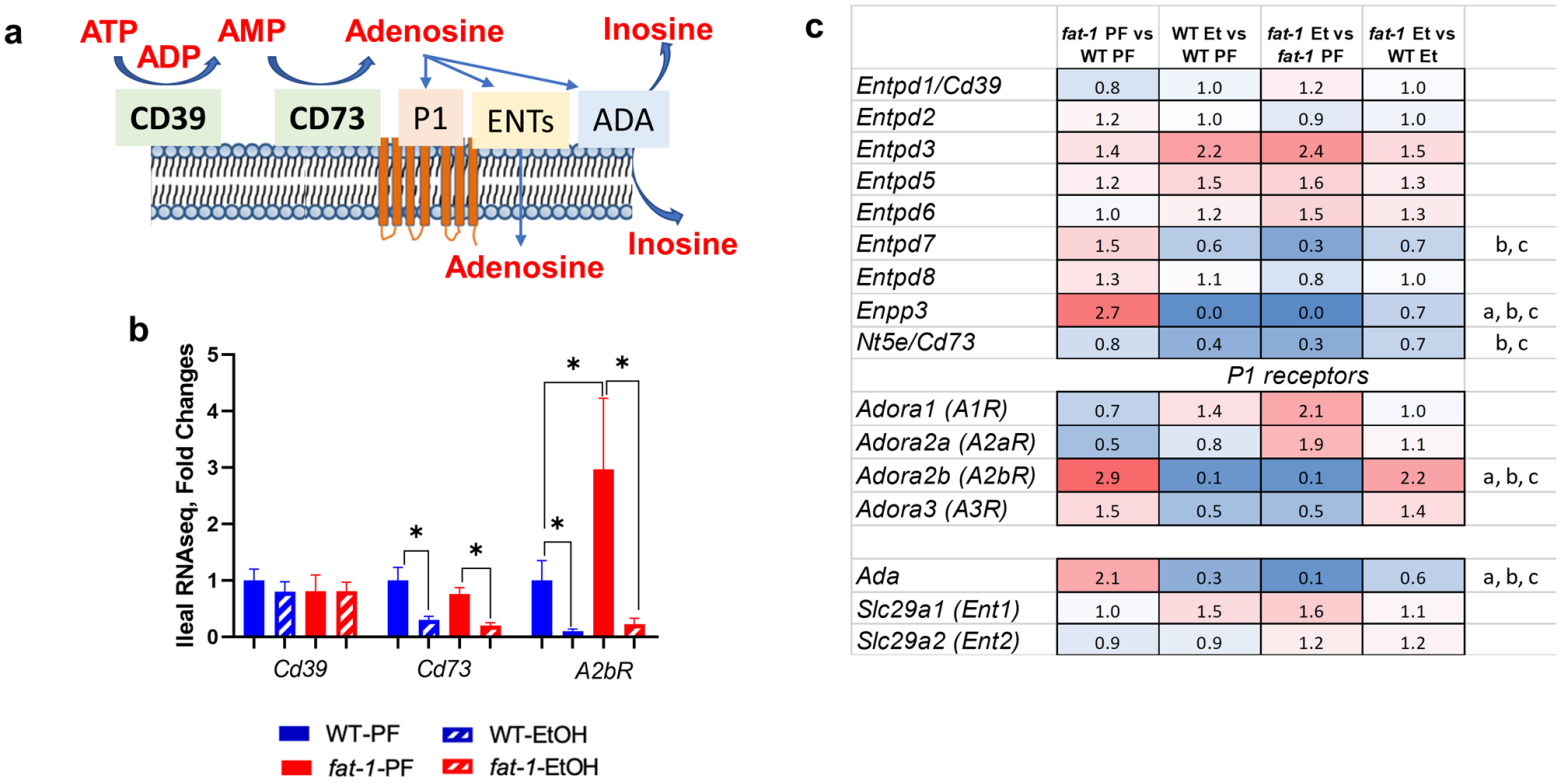
Effects of EtOH on the expression of intestinal genes involved in adenosine signaling in WT and *fat-1* mice. (**a**) Schematic of the adenosine signaling pathway. (**b**) Gene expression of *Cd39*, *Cd73*, and *A2bR* in ileal mucosa from all treatment groups. Data are expressed as fold changes vs WT PF set as 1, *p < 0.05. (**c**) Heatmap analysis of adenosine signaling genes in ileal mucosa across multiple comparisons. Data are expressed as fold changes for indicated groups. Significance for the *fat-1* PF vs WT PF comparison is denoted by (**a**), for the WT EtOH vs WT PF (**b**), for *fat-1* EtOH vs *fat-1* PF (**c**), and for *fat-1* EtOH vs WT EtOH (**d**). n = 3–5 mice per group.

Adenosine signaling is recognized as an important endogenous anti-inflammatory pathway in various diseases, including intestinal injury and inflammation^21,22^. Adenosine triphosphate (ATP) is released by stressed, apoptotic, and necrotic intestinal epithelial cells (IECs) as well as bacteria during inflammation^23^. ATP is converted to adenosine by cell surface ectonucleotidases ectonucleoside triphosphate diphosphohydrolase-1 (CD39) and ecto-5′ nucleotidase (CD73). Adenosine signals via several receptors and is inactivated by equilibrative nucleoside transporters (ENTs) or by adenosine deaminase (ADA)^24^ (Fig. 8a). RNAseq analysis in our study revealed no significant differences in *Cd39* due to EtOH consumption or genotype, while *Cd73* was significantly down-regulated by EtOH in both WT and *fat-1* mice (although the expression was not different between the *fat-1*-EtOH and WT EtOH groups) (Fig. 8b). Further, *A2bR*, a predominant adenosine receptor expressed in IECs^21,25^, was also significantly down-regulated by EtOH in both genotypes. Interestingly, *A2bR* expression was markedly higher in *fat-1* vs WT PF but not EtOH-fed animals (Fig. 8b). There were no noticeable changes in the levels of *Ent1* and *Ent2* in response to EtOH administration or genotype (Fig. 8c). *Ada* expression was significantly increased in *fat-1* PF vs WT PF mice but was down-regulated by EtOH in both genotypes (Fig. 8c). Overall, our results suggest that n3 PUFAs may enhance adenosine signaling initiating an anti-inflammatory signaling cascade in response to EtOH.

## Discussion

EtOH consumption can negatively affect intestinal health by altering intestinal homeostasis, gut microbiota, and gut barrier integrity, leading to enhanced translocation of bacteria and bacterial products which affect multiple host organs^9,26^. It has been shown that different types of dietary fats can modulate intestinal responses to chronic EtOH administration in mice^10,13^. However, the molecular mechanisms underlying these responses are not well understood. In this study we examined global intestinal transcriptome responses to chronic EtOH exposure and to increased endogenous n3 PUFA levels using WT and transgenic *fat-1* mice which endogenously biosynthesize n3 PUFAs from n6 PUFAs. We observed that WT and *fat-1* mice had both similarities and differences in intestinal transcriptional responses to EtOH suggesting that there were some conserved and unique mechanisms associated with EtOH exposure. The most prominently diminished gene caused by EtOH consumption in both genotypes was *Lct* (which was down-regulated more than 1500-fold). *Lct* is the gene encoding lactase, a digestive enzyme involved in lactose breakdown. Reduced lactase activity has been reported in humans (specifically those who drink alcohol)^27^ and in experimental animals (e.g. in rats after 3 months of EtOH consumption) likely due to direct effects of EtOH and its metabolites on the intestinal mucosa^28^. EtOH consumption also led to a pronounced down-regulation of a wide range of genes encoding drug- and xenobiotic-metabolizing enzymes (*e.g., Cyp2b10*, *Ugt2a3,* and the nuclear receptor *Car* [which regulates these genes]) in both WT and *fat-1* mice. However, it should be also noted that *Cyp2d34*, another member of cytochrome P450 family, was the most up-regulated gene in both WT and *fat-1* mice, demonstrating that EtOH significantly altered the intestinal biotransformation gene networks regardless of genotype. Among other genes that were elevated in both genotypes in response to EtOH were genes of the homeobox (*Hoxb)* family. *Hoxb* genes are transcription factors that are involved in various aspects of development and tissue regeneration, including patterning of the GI tract and differentiation of the epithelium^29^. The significance of the EtOH-induced up-regulation of *Hoxb* genes is not clear, but it may be an adaptive response to the damaging effects of EtOH on gut morphology and needs to be investigated further.

We identified gene expression patterns exclusively changed in WT or *fat-1* mice in response to EtOH challenge. There were 309 genes altered by EtOH exclusively in WT but not in *fat-1* mice. One such gene is *Fabp1* (~ 70-fold decrease in WT EtOH vs WT PF mice). *Fabp1* encodes fatty acid-binding protein 1, which is essential for proper lipid metabolism in differentiated enterocytes, and is required for enterocyte proliferation^30^, suggesting that downregulation of this gene may contribute to EtOH-induced alterations in intestinal homeostasis. We further observed that WT but not *fat-1* mice subjected to EtOH feeding exhibited reduced expression of innate immunity genes in the intestine, including *Slpi* and *Lcn2*. Intestinal SLPI has antibacterial^31^ and anti-inflammatory^32^ functions. SLPI administration has been shown to be more effective than IL-10 and TGF-β at preventing intestinal inflammation in a colitis animal model^32^. LCN2 acts on intestinal macrophages to promote bacterial clearance leading to resolution of intestinal inflammation in colitis^33^. The host response to gut bacteria is critical, and tuft cells play an important role in that process. During bacterial infection, tuft cells proliferate to elicit a pro-inflammatory response^34^. Interestingly, the expression of tuft cell markers *Sucnr1* and *Dclk* were elevated due to EtOH in WT but not *fat-1* mice. Tuft cells are also chemosensory cells that coordinate immune responses to gut derived bacteria and bacterial products^35^. Tuft cell *Sucnr1* is activated by bacterial succinate and elicits a type-2 inflammatory response in the intestine^34^. Since the expression of *Sucnr1* was increased in response to EtOH in WT but not in *fat-1* mice, the type-2 inflammatory response is likely reduced in *fat-1* mice.

One of the important observations from the current study was that *fat-1* mice exhibited greater transcriptional plasticity in response to EtOH in the intestinal mucosa. Transcriptional plasticity is considered a beneficial, adaptive response to stress^36^. Indeed, *fat-1* EtOH-fed mice had 2107 DEGs relative to *fat-1* PF mice in contrast to only 1144 DEGs observed for the WT EtOH vs WT PF mice. These diverse transcriptomic responses to EtOH in the intestines of *fat-1* mice may explain the greater protection against EtOH-induced intestinal alterations, as previously reported^14^. Of note, there were 1272 DEGs exclusively changed in *fat-1* EtOH vs *fat-1* PF mice, including down-regulation of several genes involved in cell death and cytokine signaling and up-regulation of genes involved in defense response, xenobiotic metabolism, and PPAR signaling. Of particular interest, there were several transcripts encoding pyroptosis mediators, such as *Gsdmd* and *Il18,* that were decreased in *fat-1* EtOH vs* fat-1* PF mice but not in WT EtOH vs WT PF mice. Pyroptosis is a pro-inflammatory form of cell death that has been implicated in alcoholic-liver disease^37^, but not yet in alcohol-associated gut pathology. n3 PUFAs are precursors for specialized pro-resolving mediators (SPMs, *e.g.* RvD1, RvE1)^38^. Increased tissue n3 PUFA levels in *fat-1* mice may lead to an increased pool of these SPMs^39^, which in turn may decrease the expression of pyroptosis genes in the intestinal mucosa as part of SPM-mediated resolution of inflammation. Previously, RvD1 treatment was shown to reduce pro-inflammatory cytokine levels in the ileum^14^. *Fat-1* EtOH but not WT EtOH-fed mice also exhibited diminished expression of several genes encoding pro-inflammatory cytokines and chemokines that were previously implicated in multiple intestinal pathologies, including *Cxcl16*^40^, *Il15*^41^, and *Ccl20*^42^, suggesting that n3 PUFA enrichment may dampen the intestinal inflammatory response to EtOH.

We further identified a set of genes that were differentially regulated between *fat-1* EtOH and WT EtOH-fed animals. Of interest, the expression of *Socs3* and *Ptk6* was downregulated in *fat-1* EtOH mice relative to WT EtOH mice. Diminished expression of *Socs3* is anti-inflammatory in Crohn’s disease^43^ and loss of *Ptk6* expression enhances intestinal integrity^44^. Also, many immunoglobin genes (*Igkv5-43, Igkv8-21, Igkv5-55, Igkv2-109, Igkv5-48, Igkv3-8, Igkv5-17*) were downregulated in *fat-*1 EtOH relative to WT EtOH mice. The expression of genes at the *Igkv* loci determines the variable region of light chains on antibodies that bind to antigens (and therefore the specificity of antigen recognition)^45^. Decreased expression of *Igkv* genes may indicate that there are fewer antigens present in the intestine due to n3 PUFA enrichment. These results demonstrate that n3 PUFAs may negatively regulate transcription of these pro-inflammatory mediators and change the antibody repertoire after EtOH insult. In contrast, the expression of genes relating to intestinal lipid metabolism (*Acaa1b* and *Acacb*), sugar metabolism (*Hk1*, *Akr1b8*), and amino acid metabolism (*Tph1, Tst, Cbs*) were elevated in *fat-1* EtOH relative to WT EtOH-fed mice. Preservation of metabolic gene expression may be one of the benefits of n3 PUFA enrichment after EtOH consumption.

n3 and n6 PUFAs may act as signaling molecules by activating several groups of receptors, including metabolite-sensing receptors of the FFAR and PPAR families^15,16,46^. In our study, we observed EtOH-induced expression of *Ffar2 and Ffar4* in *fat-1* but not WT mice. Since FFAR2 can positively regulate production of IL-22, a beneficial cytokine for intestinal health, as well as downstream antibacterial responses^47^, and FFAR4 activation is anti-inflammatory in IECs^16^, the up-regulation of these genes in *fat-1* mice may contribute to the beneficial effects of a n3 PUFA-enriched environment in the intestine^14^. *Pparγ*, a nuclear receptor with anti-inflammatory functions in intestinal epithelial and immune cells^48^, and a critical regulatory role in mucosal defense^49^, was up-regulated by EtOH in both WT and *fat-1* mice, most likely as an adaptive response to EtOH challenge.

Another interesting observation from our study was that the expression of several *Tlrs,* including *Tlr1/2/4/5* were induced by EtOH in both WT and *fat-1* mice. Moreover, *fat-1* EtOH-fed mice had significantly increased *Tlr2 and Tlr5* expression compared to WT EtOH-fed animals. Intestinal TLRs are important molecular regulators which protect the host from pathogens. They are involved in gut barrier function, tissue repair responses, and thus are critical for the maintenance of intestinal mucosal homeostasis. For example, it has been shown that activation of TLR2 enhanced intestinal epithelial barrier function and increased synthesis and release of IL-10, a critical anti-inflammatory cytokine^50^. Similarly, TLR5 activation by its agonist flagellin resulted in a transient transcription of genes encoding IL-17 and IL-22, which may be crucial for the early defenses against pathogen invasion of host tissues^51^. This would suggest that n3 PUFAs may enhance defense responses following an EtOH insult.

It is important to note that EtOH feeding differentially affected intestinal n6 and n3 PUFA levels in WT and *fat-1* mice. A significant EtOH-mediated increase in n6 PUFAs (with a trend for n3 PUFAs) was observed in WT but not in *fat-1* mice, suggesting that elevated levels of endogenous n6 PUFAs may also be responsible for EtOH-induced intestinal pathology in WT mice. This concept is supported by previous studies demonstrating that EtOH consumption and a diet enriched in n6 PUFA linoleic acid exacerbated intestinal inflammation and impaired gut barrier integrity and defense^8,10^. Elevated levels of various PUFAs after EtOH feeding in mice have been also reported in other organs such as the liver^52^. We do not have a definitive answer as to why there was an increase in n3 and n6-PUFAs in response to EtOH in our study, but it may be related to an overall disturbance in fatty acid metabolism, a hallmark of excessive alcohol consumption (*e.g.*, decreased fatty acid oxidation^53^). Likely, *fat-1* mice counteract the EtOH-mediated increase in n6-PUFAs through direct conversion to n3 PUFAs by the fat-1 desaturase enzyme.

Overall, our study provides a reference dataset of intestinal mucosa transcriptional responses to chronic EtOH exposure. These data will be instrumental in the formulation of specific hypotheses for follow-up studies potentially leading to new therapeutic interventions for intestinal pathological changes induced by EtOH. The study also demonstrated several favorable changes in the intestinal transcriptional repertoire in an n3 PUFA-enriched environment, which may ameliorate the deleterious effects of EtOH on intestinal homeostasis.

## Methods

### Study design

The study was approved by and performed in accordance with the guidelines of the University of Louisville Institutional Animal Care and Use Committee. The mice were handled and treated as previously described^14^. Male wild type (WT) C57Bl/6 mice and *fat-1* littermates on a C57BL/6 background were used for this study. The *fat-1* transgenic mouse line was obtained from Dr. J. X. Kang (Harvard Medical School) and has been described previously^54^. These mice express the n3 fatty acid desaturase gene *fat-1*, from *C. elegans*, in all tissues, leading to a direct conversion of n6 PUFAs to n3 PUFAs with a subsequent decrease in the n6/n3 PUFA ratio^54^. For the current study, the experimental mice were generated at the University of Louisville animal facility by breeding *fat-1* heterozygotes to WT C57BL/6 mice to obtain *fat-1* heterozygous and WT littermates. All mice were genotyped at weaning. Our pilot studies and reports from others^54,55^ confirmed that *fat-1* mice exhibited elevated n3 PUFA levels and decreased n6/n3 PUFA ratio in various tissues. Expression of the *fat-1* gene was validated by standard PCR (forward primer: 5′-CGGTTTCTGCGATGGATCCCAC-3′, reverse primer: 5′-CACAGGAACCGGGCAAAGAA-3′) on tail biopsies. The gene *Gdf-5* was used as a positive control (forward primer: 5′-AAGCCCTCAGTCAGTTGTGC-3′, reverse primer: 5′-AAAACCATGAAAGGAGTGGG-3′). See Supplemental Fig. S2a,b for breeding scheme and representative results of genotype conformation by PCR, respectively. The 8–10 week old male WT and *fat-1* littermates were placed on control or EtOH-containing Lieber-DeCarli liquid diets (BioServ, Flemington, NJ). The mice were fed for 6 weeks with a stepwise increase in EtOH concentration (0%, 1%, and 2% for two days each, 4% and 5% for one week each and then 6% for 3 weeks, see Supplemental Fig. S1a for experimental model). The control pair-fed mice (PF) received and consumed the same amount of isocaloric food (as determined by caloric intake with maltose dextrin substituting for EtOH) that EtOH-fed animals consumed in the previous day (pair-feeding protocol). Food consumption was recorded daily and body weights weekly. PF mice consumed the full amount of food provided each day (similar as EtOH-fed animals); the average daily food consumption by weeks is reported in Supplemental Fig. S1b. At the end of the feeding protocol, the mice were anesthetized with ketamine/xylazine (100/20 mg/kg) and euthanized by exsanguination and thoracotomy.

### Intestinal tissue isolation and RNA preparation

After euthanasia, the intestines were removed, and the ileum was excised and thoroughly flushed with PBS. An approximately 1.5 cm segment of the terminal portion of the ileum was isolated and snap-frozen in liquid nitrogen, then stored at − 80 °C. See Supplemental Fig. S3 for the location of ileal segment used for RNA isolation. Total ileum RNA was isolated using Trizol reagent (Thermo Fisher, Waltham, MA) from 3–5 mice/group followed by removal of contaminating genomic DNA by digestion with DNase I (TURBO DNA-free kit, Thermo Fisher). RNA was further purified and concentrated using the GeneJET RNA cleanup and concentration micro kit (Thermo Fisher). RNA integrity was determined by the Agilent Bioanalyzer 2100 (Agilent, Santa Clara, CA). Only RNA samples with integrity values ranging from 7 to 9 were used for RNASeq analysis.

### Intestinal tissue RNA-sequencing and bioinformatics

The transcriptome sequencing library preparation and sequencing was performed by the University of Louisville Center for Genetics in Molecular Medicine core facility using the TruSeq Stranded mRNA library prep kit (part no. 20020594). The full details for RNA Sequencing have been described previously^14^, but pertinent methodology is described below. Briefly, poly A purification and RNA fragmentation were performed on total RNA and Superscript II (Thermo-Fisher) was used to generate cDNA, with subsequent removal of the RNA template. Second strand synthesis was performed with dUTP to ensure stranded libraries, and double-stranded cDNA were purified with AMPure XP beads. 3′-Ends were adenylated, indexing adapters were ligated onto the ends, and libraries were once again purified with AMPure beads. Libraries were loaded onto an Agilent DNA 1000 chip and validated on an Agilent 2100 Bioanalyzer. Quantitation was performed with the Illumina Library Quantification Kit (Illumina Inc., San Diego, CA), ABI Prism qPCR Mix from Kapa Biosystems (Wilmington, MA). Three dilutions were tested in triplicate. Libraries were diluted to 10 nM, pooled, further diluted and denatured and analyzed on a NextSeq 500 v2 150 cycles High Output kit (cat#FC-404-2002) in a 2 × 75 bp PE.

### RNA-seq bioinformatic and statistical analysis

Analysis was conducted by the NIH-funded Kentucky Biomedical Research Infrastructure Network Bioinformatics Core. The full details for this method have been published previously^14^, but pertinent details are described below. Sequences were directly aligned to the *Mus musculus* reference genome assembly (mm10.fa) using tophat2 (version 2.0.13)^56^ guided by Ensembl build 82 mouse transcripts. To confirm expression of the *fat-1* transgene, we aligned each of the original reads to the *C. elegans* genome (ce10). A small fraction of the reads (less than 1%) mapped to *C. elegans*, as expected. We then extracted the reads from the region of the *fat-1* gene and constructed a UCSC genome browser track for these regions. Differentially expressed genes (DEGs) between experimental groups were identified using the Tuxedo suite of programs including cuffdiff2 (version 2.2.1)^57,58^. A *p*-value cutoff ≤ 0.05, *q*-value cutoff ≤ 0.05 was used to determine differential expression. The full list of significant DEGs for each comparison can be found in Supplemental Materials Excel File S1. DEGs with a cutoff of ≥ two-fold change were used for further pairwise comparisons in Volcano Plots, GO Processes, and STRING Cluster Analysis. This threshold was used to obtain a higher magnitude of differences, and to identify more robust gene expression changes and the accompanying biological processes.

Cytoscape STRING plugin was used for visualizing molecular interactions between DEGs and identifying Gene Ontology:Biological Processes (GO:BPs) and Kyoto Encyclopedia of Genes and Genomes (KEGG) Pathways that include the DEGs^59^. Cytoscape analysis identified enriched GO:BPs (representative of DEGs increased) and diminished GO:BPs (representative of DEGs decreased) for their respective comparisons. The top ten enriched and diminished GO:BPs were reported (if 10 were identified) along with their associated false-discovery rate (FDR < 0.05). Similarly, Search Tool for the Retrieval of Interacting Genes/Proteins (STRING) Analysis and gene clustering were conducted in CytoScape followed by KEGG Pathway enrichment (FDR < 0.05). Gene clusters and their associated KEGG Pathway were used for DEGs increased and decreased in the respective comparisons.

STRING cluster diagrams list gene clusters by biological pathways they represent. Genes with connections as determined by STRING analysis are within the same circle, and the associated process is listed near the cluster. Clusters with purple circles and green nodes were decreased for the respective comparison, while clusters with yellow circles and copper nodes were increased.

### Measurement of n3 and n6 PUFAs

Fatty acids were measured in the intestines of the same mice as used in the RNAseq analysis. The assay was conducted at the Laboratory for Lipid Medicine and Technology at Massachusetts General Hospital and Harvard University according to a well-established protocol^60^ with slight modifications. Briefly, frozen ileum segments were ground under liquid nitrogen using a mortar and pestle. The samples were then processed for lipid extraction and methylation by adding 1:1 hexane and 14% boron trifluoride/methanol and heated at 100 °C for one hour. Fatty acid methyl esters were analyzed on a fully automated 6890 N Network Gas Chromatography Instrument equipped with a flame-ionization detector (Agilent Technologies, Palo Alto, CA). Individual fatty acid concentrations were determined by retention time using a reference standard, GLC461, and quantitated by a reference to an internal standard, C13:0 FAME (Nu-Chek Prep, Elysian, MN).

## Supplementary information

Supplementary Information

Supplementary Information 2

## Data Availability

The GEO accession number for the RNAseq data reported in this paper is GSE133253. All other data generated or analyzed during this study are included in this published article and its supplementary information file.
