# Supplementary material for: Transcriptional signatures of the small intestinal mucosa in response to ethanol in transgenic mice rich in endogenous n3 fatty acids
Source: Sci Rep. 2020 Nov 16;10:19930. doi: 10.1038/s41598-020-76959-6 (PMC7670449; doi:10.1038/s41598-020-76959-6)
Supplement: Supplementary file 2 — Supplementary Information 2 [file 41598_2020_76959_MOESM2_ESM.pdf]

Supplemental Information

Transcriptional signatures of the small intestinal mucosa in response to ethanol in transgenic mice rich in endogenous n3 fatty acids

Josiah E. Hardesty, Jeffrey B. Warner, Ying L. Song, Eric C. Rouchka, Chih-Yu Chen, Jing X. Kang, Craig J. McClain, Dennis R. Warner, and Irina A. Kirpich

Supplemental Figures:

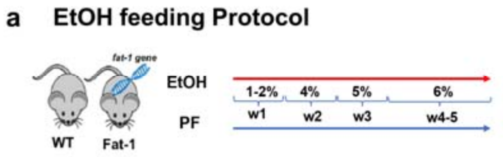

b Food Consumption

	Week 1 ml/day	Week 2 ml/day	Week 3 ml/day	Week 4 ml/day	Week 5 ml/day	Week 6 ml/day
WT (PF and EtOH)	16.6 ± 0.6	16.6 ± 0.5	15.0 ± 0.3	13.5 ± 0.3	14.3 ± 0.5	14.4 ± 0.5
fat-1 (PF and EtOH)	15.1 ± 0.9	16.3 ± 0.9	15.9 ± 0.6	13.0 ± 0.6	14.6 ± 0.7	14.8 ± 0.4

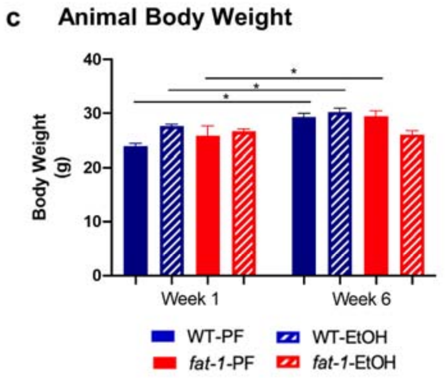

d Intestinal PUFA analysis

	n6	n3	n6/n3
WT PF	7.10 ± 0.92	0.48 ± 0.04 *	14.76 ± 1.10 *
WT EtOH	15.38 ± 1.89 ^ #	0.75 ± 0.05	20.45 ± 2.02 ^
fat-1 PF	8.67 ± 1.34	1.19 ± 0.25	8.35 ± 1.74
fat-1 EtOH	9.36 ± 1.77	1.26 ± 0.04	7.4 ± 1.28

P < 0.05; * WT PF vs *fat-1* PF; ^ WT EtOH vs *fat-1* EtOH; # WT PF vs WT EtOH

Supplemental Figure S1. Experimental Design and Metabolic Parameters. (a) EtOH feeding model. **(b)** Average food consumption by experimental animals. **(c)** Beginning and ending body weights of PF and EtOH-fed WT and *fat-1* mice. **(d)** Ileum n3 and n6 PUFA levels demonstrating that *fat-1* mice have elevated n3 PUFAs relative to WT mice. Data are presented as mean ± SEM. * p < 0.05. n=3-5 mice per group.

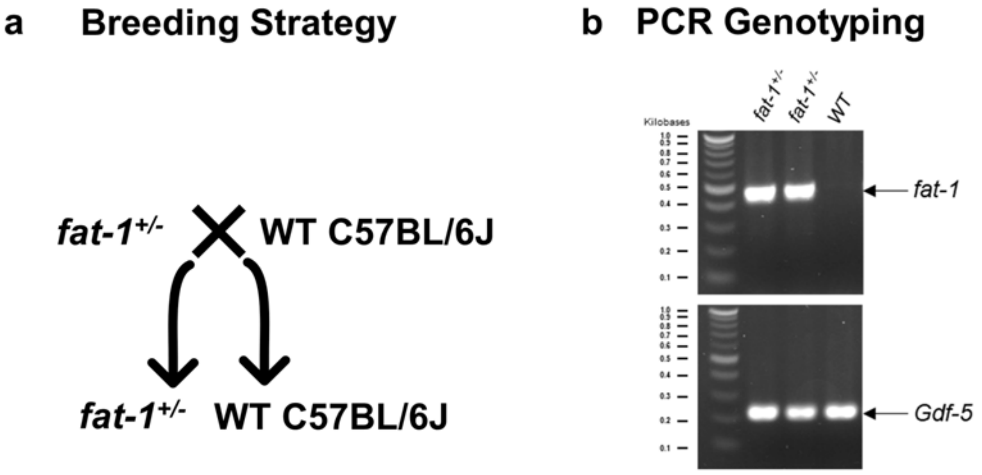

Supplemental Figure S2. Description of the *fat-1* mice. (a) Breeding strategy to develop *fat-1*$^{+/-}$ heterozygous mice and WT littermates. (**b**) Genotype confirmation by PCR and gel electrophoresis. The *fat-1* PCR product is only present in samples from *fat-1* mice whereas *Gdf-5* is present in all samples (positive control) in the representative gel.

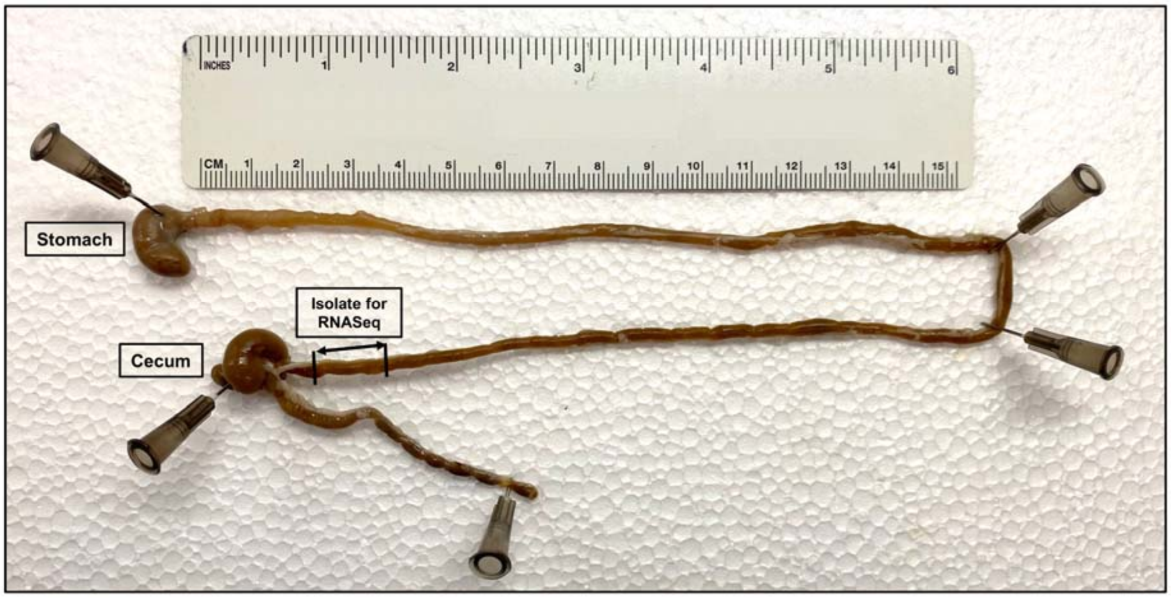

Supplemental Figure S3. Mouse ileum isolation. Layout of mouse gastrointestinal tract identifying the segment of intestine isolated for ileum RNASeq.

Supplemental Tables:

Supplementary Table S1. List of the top 10 up-regulated and down-regulated genes (by fold-change) in the intestinal mucosa in *fat-1* compared to WT pair-fed control mice (selected from 420 up- and 210 down-regulated genes).

**Identifier**	**Gene title**	**Fold change**	***p*-value**	***q*-value**
ENSMUSG00000048473	*Gm766*	40.6	0.00005	0.0022175
ENSMUSG00000045991	*Onecut2*	32.47	0.00005	0.0022175
ENSMUSG00000059654	*Reg1*	20.7	0.00005	0.0022175
ENSMUSG00000076540	*Igkv4-80*	16.8	0.00005	0.0022175
ENSMUSG00000046213	*Cym*	15.2	0.00005	0.0022175
ENSMUSG00000095429	*Ighv5-12*	12.0	0.0006	0.016949
ENSMUSG00000054932	*Afp*	10.2	0.00005	0.0022175
ENSMUSG00000076536	*Igkv4-86*	9.3	0.00005	0.0022175
ENSMUSG00000036853	*Mcoln3*	9.0	0.00005	0.0022175
ENSMUSG00000067235	*H2-Q10*	8.8	0.00005	0.0022175
ENSMUSG00000074442	*Defa-rs7*	-26.4	0.00005	0.0022175
ENSMUSG00000094662	*Gm21498*	-19.1	0.00005	0.0022175
ENSMUSG00000094993	*Igkv4-51*	-15.0	0.00035	0.011114
ENSMUSG00000031089	*Slc6a14*	-11.9	0.00005	0.0022175
ENSMUSG00000104452	*Ighv8-8*	-11.4	0.00005	0.0022175
ENSMUSG00000096844	*Igkv6-14*	-8.6	0.00085	0.0222623
ENSMUSG00000026109	*Tmeff2*	-6.9	0.0005	0.0147833
ENSMUSG00000033847	*Pla2g4c*	-6.4	0.00005	0.0022175
ENSMUSG00000078706	*Gm53*	-5.7	0.00005	0.0022175
ENSMUSG00000094198	*Ighv1-50*	-5.6	0.00005	0.0022175

Supplementary Table S2. List of up-regulated and down-regulated genes in the intestinal mucosa in WT EtOH compared to WT pair-fed control mice. The top 10 up-regulated and down-regulated genes (by fold-change) are shown in a list (selected from 481 up- and 663 down-regulated genes).

Identifier	Gene title	Fold change	*p*-value	*q*-value
ENSMUSG00000032122	*Slc37a2*	47.2	0.00005	0.00128109
ENSMUSG00000073608	*Gm6086*	39.1	0.00005	0.00128109
ENSMUSG00000094559	*Cyp2d34*	29.5	0.0001	0.00236173
ENSMUSG00000057074	*Ces1g*	21.1	0.00005	0.00128109
ENSMUSG00000001943	*Vsig2*	20.4	0.00155	0.0230212
ENSMUSG00000033847	*Pla2g4c*	19.6	0.00005	0.00128109
ENSMUSG00000022650	*Retnlb*	18.2	0.00005	0.00128109
ENSMUSG00000068893	*Sprr2a2*	17.2	0.00005	0.00128109
ENSMUSG00000076666	*Ighv14-4*	15.2	0.00005	0.00128109
ENSMUSG00000096672	*Ighv1-63*	14.1	0.0009	0.015004
ENSMUSG00000026354	*Lct*	-1791.5	0.00005	0.00128109
ENSMUSG00000021944	*Gata4*	-135.6	0.00005	0.00128109
ENSMUSG00000029134	*Plb1*	-130.9	0.00005	0.00128109
ENSMUSG00000024354	*Slc23a1*	-93.3	0.0022	0.0307049
ENSMUSG00000035780	*Ugt2a3*	-79.9	0.00005	0.00128109
ENSMUSG00000054422	*Fabp1*	-69.8	0.00005	0.00128109
ENSMUSG00000027690	*Slc2a2*	-43.2	0.00005	0.00128109
ENSMUSG00000030483	*Cyp2b10*	-40.5	0.00005	0.00128109
ENSMUSG00000076655	*Ighv4-1*	-35.3	0.00005	0.00128109
ENSMUSG00000005980	*Dnase1*	-33.7	0.00005	0.00128109

Supplementary Table S3. List of up-regulated and down-regulated genes in the intestinal mucosa in *fat-1* EtOH compared to *fat-1* pair-fed control mice. The top 10 up-regulated and down-regulated genes are shown in a list (by fold-change) (selected from 1045 up- and 1062 down-regulated genes).

Identifier	Gene title	Fold change	*p*-value	*q*-value
ENSMUSG00000027556	*Car1*	1454.1	0.00345	0.027756
ENSMUSG00000094559	*Cyp2d34*	258.4	0.00315	0.0258838
ENSMUSG00000026390	*Marco*	151.1	0.00135	0.0136846
ENSMUSG00000022650	*Retnlb*	131.0	0.0014	0.0140118
ENSMUSG00000031089	*Slc6a14*	105.5	0.00005	0.00092057
ENSMUSG00000037362	*Nov*	96.6	0.00005	0.00092057
ENSMUSG00000033579	*Fa2h*	87.0	0.00005	0.00092057
ENSMUSG00000013653	*1810065E05Rik*	71.2	0.00005	0.00092057
ENSMUSG00000096001	*2610528A11Rik*	47.3	0.00005	0.00092057
ENSMUSG00000024039	*Cbs*	42.6	0.00005	0.00092057
ENSMUSG00000026354	*Lct*	-1668.9	0.00005	0.00092057
ENSMUSG00000030483	*Cyp2b10*	-274.6	0.00005	0.00092057
ENSMUSG00000035780	*Ugt2a3*	-217.1	0.00005	0.00092057
ENSMUSG00000021944	*Gata4*	-191.2	0.00005	0.0210196
ENSMUSG00000029134	*Plb1*	-166.9	0.00005	0.00092057
ENSMUSG00000002992	*Apoc2*	-151.5	0.00005	0.00092057
ENSMUSG00000074373	*Gm10680*	-120.0	0.00045	0.0058381
ENSMUSG00000036216	*Leap2*	-115.0	0.00005	0.00092057
ENSMUSG00000032080	*Apoa4*	-106.5	0.00335	0.0270893
ENSMUSG00000019989	*Enpp3*	-106.3	0.00005	0.00092057

Supplementary Table S4. List of commonly up-regulated and down-regulated genes in the intestinal mucosa in *fat-1* and WT mice in response to EtOH. The top 10 up-regulated and down-regulated genes (by fold-change) are shown in a list (selected from 310 up- and 525 down-regulated genes).

		***Fat-1* EtOH vs *fat-1* PF**			**WT EtOH vs WT PF**		
**Identifier**	**Gene title**	**fold**	***p*-value**	***q*-value**	**fold**	***p*-value**	***q*-value**
ENSMUSG00000094559	*Cyp2d34*	258.4	0.00315	0.0258838	29.5	0.0001	0.00236173
ENSMUSG00000022650	*Retnlb*	131.0	0.0014	0.0140118	18.2	0.00005	0.00128109
ENSMUSG00000031089	*Slc6a14*	105.5	0.00005	0.00092057	6.7	0.00005	0.00128109
ENSMUSG00000033579	*Fa2h*	87.0	0.00005	0.00092057	8.3	0.00005	0.00128109
ENSMUSG00000013653	*1810065E05Rik*	71.2	0.00005	0.00092057	8.3	0.00005	0.00128109
ENSMUSG00000096001	*2610528A11Rik*	47.3	0.00005	0.00092057	3.8	0.00005	0.00128109
ENSMUSG00000024039	*Cbs*	42.6	0.00005	0.00092057	4.6	0.00005	0.00128109
ENSMUSG00000027870	*Hao2*	38.9	0.00005	0.00092057	8.4	0.00005	0.00128109
ENSMUSG00000027875	*Hmgcs2*	35.2	0.00005	0.00092057	9.1	0.00005	0.00128109
ENSMUSG00000026811	*St6galnac6*	32.3	0.00005	0.00092057	4.5	0.00005	0.00128109
ENSMUSG00000026354	*Lct*	-1668.9	0.00005	0.00092057	-1791.6	0.00005	0.00128109
ENSMUSG00000030483	*Cyp2b10*	-274.6	0.00005	0.00092057	-40.5	0.00005	0.00128109
ENSMUSG00000035780	*Ugt2a3*	-217.1	0.00005	0.00092057	-79.9	0.00005	0.00128109
ENSMUSG00000021944	*Gata4*	-191.2	0.0024	0.0210196	-135.6	0.00005	0.00128109
ENSMUSG00000029134	*Plb1*	-166.9	0.00005	0.00092057	-130.9	0.00005	0.00128109
ENSMUSG00000002992	*Apoc2*	-151.5	0.00005	0.00092057	-19.9	0.00005	0.00128109
ENSMUSG00000074373	*Gm10680*	-120.0	0.00045	0.0058381	-20.6	0.00025	0.00525227
ENSMUSG00000036216	*Leap2*	-115.0	0.00005	0.00092057	-11.2	0.00005	0.00128109
ENSMUSG00000032080	*Apoa4*	-106.5	0.00335	0.0270893	-20.5	0.00005	0.00128109
ENSMUSG00000019989	*Enpp3*	-106.3	0.00005	0.00092057	-25.2	0.00005	0.00128109

Supplementary Table S5. List of genes exclusively up-regulated and down-regulated in the intestinal mucosa in WT mice in response to EtOH. The top 10 up-regulated and down-regulated genes (by fold-change) are shown in a list (selected from 171 up- and 138 down-regulated genes).

**Identifier**	**Gene title**	**Fold change**	***p*-value**	***q*-value**
ENSMUSG00000032122	*Slc37a2*	47.2	0.00005	0.00128109
ENSMUSG00000076666	*Ighv14-4*	15.2	0.00005	0.00128109
ENSMUSG00000096672	*Ighv1-63*	14.1	0.0009	0.015004
ENSMUSG00000050296	*Abca12*	13.2	0.00005	0.00128109
ENSMUSG00000086474	*9130204K15Rik*	11.6	0.0003	0.00615377
ENSMUSG00000065037	*Rn7sk*	11.0	0.00025	0.00525227
ENSMUSG00000098814	*Igkv19-93*	6.7	0.00005	0.00128109
ENSMUSG00000079174	*Gm3054*	6.1	0.00005	0.00128109
ENSMUSG00000095204	*Ighv1-52*	5.7	0.00005	0.00128109
ENSMUSG00000086389	*Gm15998*	5.2	0.0015	0.0224225
ENSMUSG00000054422	*Fabp1*	-69.8	0.00005	0.00128109
ENSMUSG00000094164	*Ighv2-3*	-16.1	0.00005	0.00128109
ENSMUSG00000094993	*Igkv4-51*	-15.1	0.00075	0.0130517
ENSMUSG00000014351	*Gip*	-14.3	0.00005	0.00128109
ENSMUSG00000104452	*Ighv8-8*	-8.2	0.00005	0.00128109
ENSMUSG00000032454	*Rbp2*	-7.7	0.00005	0.00128109
ENSMUSG00000102888	*Ighv1-11*	-7.5	0.00035	0.00701364
ENSMUSG00000076538	*Igkv13-84*	-7.4	0.0005	0.00940854
ENSMUSG00000076532	*Igkv4-91*	-6.9	0.00005	0.00128109
ENSMUSG00000029082	*Bst1*	-6.5	0.00005	0.00128109

Supplementary Table S6. List of genes exclusively up-regulated and down-regulated in the intestinal mucosa in *fat-1* mice in response to EtOH. The top 10 up-regulated and down-regulated genes (by fold-change) are shown in a list (selected from 735 up- and 537 down-regulated genes).

**Identifier**	**Gene title**	**Fold change**	***p*-value**	***q*-value**
ENSMUSG00000027556	*Car1*	1454.1	0.00345	0.027756
ENSMUSG00000026390	*Marco*	151.1	0.00135	0.0136846
ENSMUSG00000037362	*Nov*	96.6	0.00005	0.00092057
ENSMUSG00000032068	*Plet1*	30.9	0.00005	0.00092057
ENSMUSG00000021803	*Cdhr1*	29.8	0.00125	0.0128913
ENSMUSG00000048337	*Npy4r*	26.6	0.0009	0.0100225
ENSMUSG00000046589	*Lrrc8e*	17.8	0.0009	0.0100225
ENSMUSG00000032028	*Nxpe2*	14.9	0.00005	0.00092057
ENSMUSG00000017723	*Wfdc2*	14.8	0.00005	0.00092057
ENSMUSG00000029088	*Kcnip4*	14.5	0.00035	0.00481741
ENSMUSG00000041660	*Bbox1*	-58.5	0.00005	0.00092057
ENSMUSG00000068323	*Slc4a5*	-25.4	0.00005	0.00092057
ENSMUSG00000005237	*Dnah2*	-19.8	0.00005	0.00092057
ENSMUSG00000002324	*Rec8*	-18.5	0.00005	0.00092057
ENSMUSG00000054932	*Afp*	-15.5	0.0027	0.0230512
ENSMUSG00000095981	*Ighv10-1*	-14.4	0.00005	0.00092057
ENSMUSG00000038496	*Slc19a3*	-14.3	0.0001	0.00170036
ENSMUSG00000076665	*Ighv7-1*	-13.7	0.00005	0.00092057
ENSMUSG00000030402	*Ppm1n*	-10.9	0.0013	0.0132723
ENSMUSG00000070332	*Trim80*	-9.8	0.0006	0.00729846

Supplementary Table S7. List of top 10 up-regulated and down-regulated genes (by fold-change) in the intestinal mucosa in *fat-1* EtOH compared to WT EtOH littermates (selected from 80 up- and 14 down-regulated genes).

**Identifier**	**Gene title**	**Fold change**	***p*-value**	***q*-value**
ENSMUSG00000037362	*Nov*	60.6	0.00005	0.00367576
ENSMUSG00000032068	*Plet1*	46.3	0.00005	0.00367576
ENSMUSG00000065987	*Cd209b*	19.5	0.00005	0.00367576
ENSMUSG00000076674	*Ighv1-85*	15.7	0.00005	0.00367576
ENSMUSG00000039579	*Grin3a*	13.6	0.00005	0.00367576
ENSMUSG00000032028	*Nxpe2*	10	0.00005	0.00367576
ENSMUSG00000094806	*Cyp2d10*	9.6	0.00015	0.0282662
ENSMUSG00000055210	*Foxd2*	8.9	0.0002	0.0341412
ENSMUSG00000095457	*Gm8989*	7.7	0.00005	0.0117016
ENSMUSG00000019775	*Rgs17*	6.9	0.0002	0.0341412
ENSMUSG00000096150	*Ighv3-8*	-18.9	0.00005	0.00367576
ENSMUSG00000076666	*Igkv2-109*	-10.8	0.00005	0.00367576
ENSMUSG00000076563	*Igkv5-48*	-7.5	0.00005	0.0117016
ENSMUSG00000033847	*Pla2g4c*	-7.0	0.00005	0.0117016
ENSMUSG00000031073	*Fgf15*	-6.8	0.00005	0.0117016
ENSMUSG00000096833	*Igkv4-55*	-5.9	0.0002	0.0341412
ENSMUSG00000076586	*Igkv8-21*	-4.7	0.00005	0.0117016
ENSMUSG00000095571	*Ighv5-17*	-4.5	0.00005	0.0117016
ENSMUSG00000094433	*Igkv5-43*	-3.1	0.00005	0.0117016
ENSMUSG00000020826	*Nos2*	-2.9	0.00005	0.0117016
